# B-Scan and “En-Face” Spectral-Domain Optical Coherence Tomography Imaging for the Diagnosis and Followup of Acute Retinal Pigment Epitheliitis

**DOI:** 10.1155/2013/260237

**Published:** 2013-02-18

**Authors:** Flore De Bats, Benjamin Wolff, Martine Mauget-Faÿsse, Claire Scemama, Laurent Kodjikian

**Affiliations:** ^1^Department of Ophthalmology Croix-Rousse University Hospital and Hospices Civils de Lyon, University of Medicine Lyon 1, 103 Grande Rue de la Croix-Rousse, 69317 Lyon Cedex 04, France; ^2^Sahel Department, Rothschild Ophthalmologic Foundation, 25 Rue Manin, 75019 Paris, France; ^3^Kleber Retinal Center, 50 Cours Franklin Roosevelt, 69006 Lyon, France

## Abstract

*Purpose*. To report B-scan and “En-face” spectral-domain optical coherence tomography (SD-OCT) findings in acute retinal pigment epitheliitis (ARPE). *Methods*. Two patients (3 eyes) with ARPE were examined. Fluorescein and indocyanine green (ICGA) angiography, B-scan, and “En-face” SD-OCT were performed in each patient at initial and follow-up visits. *Results*. Both patients presented with acute onset of blurred vision, and one with bilateral involvement. B-can OCT revealed disruption of the macular retinal pigment epithelial (RPE) inner band layer and photoreceptors' inner and outer segment (IS-OS) junction. Hyperreflective dots were observed in the outer nuclear layer (ONL) above the RPE/IS-OS disruption. Just around these hyperreflective dots, slight thickening of the hyperreflective IS/OS junction was observed. During the late phase, indocyanine green angiography (ICGA) showed a macular cockade-like hyperfluorescent halo. “En-face” OCT showed the same cockade-like appearance with a hyporeflective center and a hyperreflective border matching the pattern observed on ICGA. At followup, as vision improved without treatment, B-scan OCT demonstrated progressive resolution of the hyperreflective and disrupted lesions; “en-face” OCT also showed disappearance of the macular cockade-like halo with a transient discrete hyperreflective macular star at the RPE level in one eye. *Conclusion*. “En-face” OCT associated with B-scan SD-OCT analysis appears to be very helpful in the diagnosis and followup of ARPE. The pathophysiology of ARPE remains complex and still poorly understood. These techniques help define the location and extent of structural damage occurring in this disease.

## 1. Introduction

Acute retinal pigment epitheliitis (ARPE), or Krill's disease, named after its first description by Krill in 1974, is a benign, self-limited, bilateral yet asymmetrical pathology affecting adults between 10 and 40 years of age [1]. It is characterized by acute visual blurring which usually resolves, without treatment, within a few weeks. The acute occurrence of this disease after flu-like symptoms has suggested that a viral infection could be the origin of ARPE [2, 3]. 

In addition to these clinical parameters, fluorescein (FA) and indocyanine green angiography (ICGA) help in identifying the diagnosis. The high resolution of the scans generated by spectral-domain optical coherence tomography (SD-OCT) offers a helpful tool in the management of ARPE. SD-OCT allows a direct, noncontact visualization of the involved retinal layers: inner segment-outer segment (IS-OS) junction and inner band of the retinal pigment epithelium (RPE).

“En-face” OCT is a novel imaging technique generating frontal scans derived from SD-OCT [4]. In contrast, B-scans for conventional OCT are derived from sagittal and transverse sections. The use of “en-face” OCT for ARPE allows a frontal, layer-by-layer visualization of the retina. In this study, the results from SD-OCT B-scans and “en-face” OCT were compared with data from classical retinal imaging, namely, fundus photography and angiography [5]. 

## 2. Methods

Two patients with ARPE lesions underwent complete ocular examination comprising best-corrected visual acuity (BCVA), biomicroscopy, fundoscopy, autofluorescence, fluorescein angiography (FA), and indocyanine green angiography (ICGA). SD-OCT B-scans and “en-face” OCT were performed at the initial visit and during the follow-up period with a Spectralis-HRA (Spectralis HRA+OCT; Heidelberg Engineering, Heidelberg, Germany) and a Cirrus OCT (Carl Zeiss Meditec, Dublin, CA, USA). 

## 3. Results

### 3.1. Patient 1

**Figure 1:**
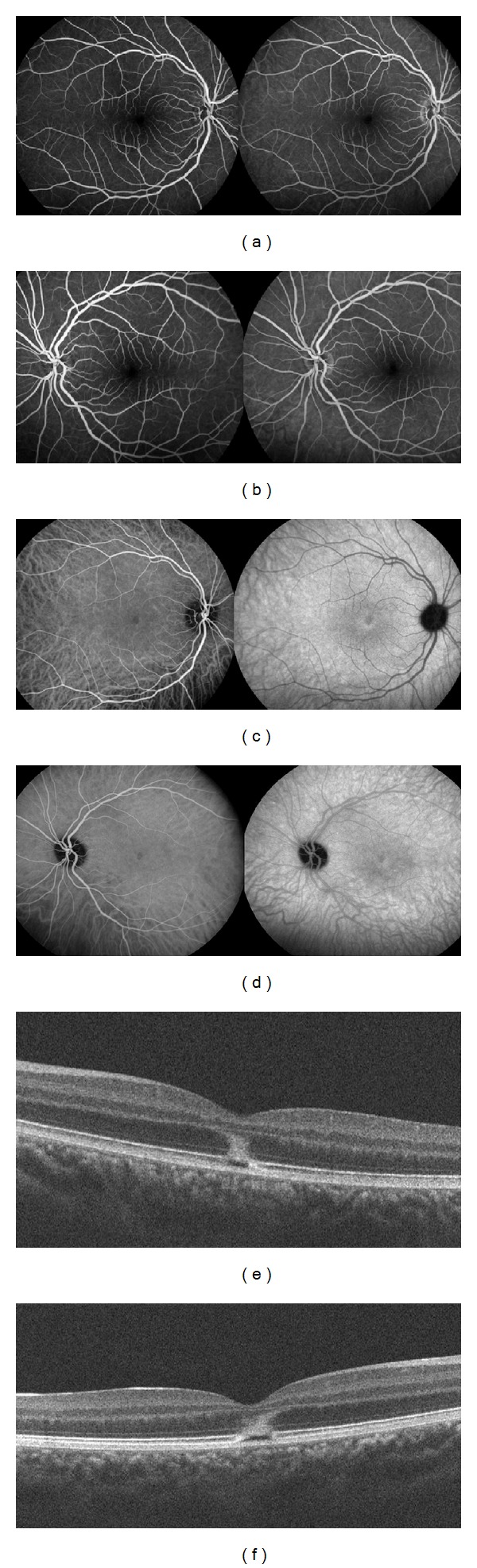
Patient 1. (a), (c), (e): Right eye. (b), (d), (f) Left eye. (a), (b). Fluorescein angiography (2 and 5 minutes), at the initial visit, showing no hyperfluorescence. (c), (d). Indocyanine green angiography (5 and 18 minutes), at the initial visit, showing a hyperfluorescent halo in the macular area. (e), (f) Spectral-domain optical coherence tomography (B-scan), at the initial visit, showing disruptions of the IS/OS junction and the RPE inner band. There is a large hyperreflective lesion involving the external nuclear layer.

**Figure 2:**
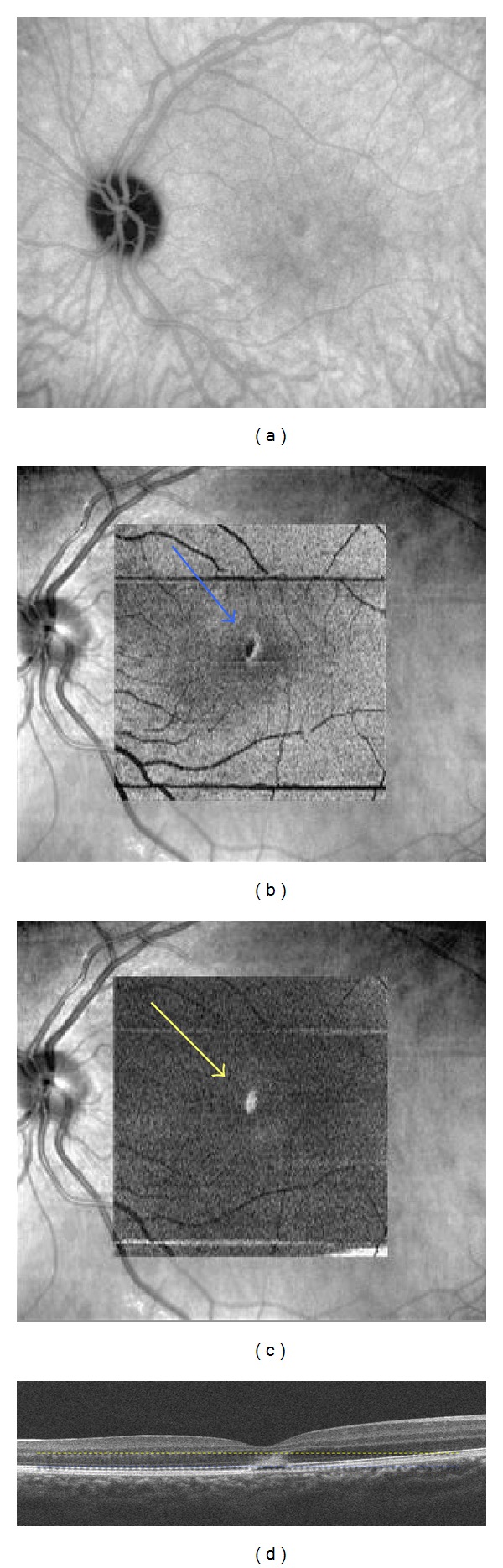
Patient 1, left eye. (a) Indocyanine green angiography (18 minutes), at the initial visit, showing a cockade-like lesion in the macular area at the level of the IS-OS junction. (b) “En-face” spectral-domain optical coherence tomography showing a central cockade-like lesion with a hyporeflective center and a hyperreflective border (blue arrow). (c) “En-face” OCT showing a hyperreflective punctuate lesion located in the outer nuclear layer (yellow arrow). (d) B-scan spectral-domain optical coherence tomography showing hyperreflective edges of the disrupted lesion corresponding with the cockade-like lesion and a hyperreflective lesion in the outer nuclear layer corresponding with the central hyperreflective point on “en-face” OCT (yellow and blue lines).

**Figure 3:**
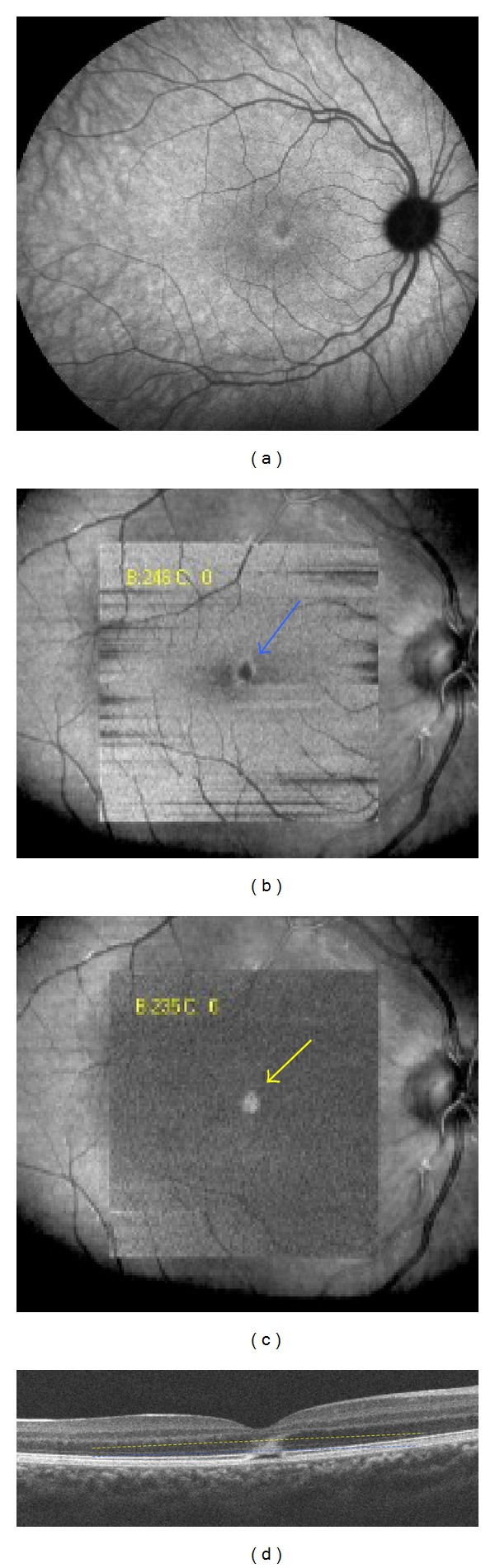
Patient 1, right eye. (a) Indocyanine green angiography (18 minutes), at the initial visit, showing a cockade-like lesion in the macular area at the level of the IS-OS junction. (b) “En-face” spectral-domain optical coherence tomography showing a central cockade-like lesion with a hyporeflective center and a hyperreflective border (blue arrow). (c) “En-face” OCT showing a hyperreflective punctuate lesion located in the outer nuclear layer (yellow arrow). (d) B-scan spectral-domain optical coherence tomography showing hyperreflective edges of the disrupted lesion corresponding with the cockade-like lesion and a hyperreflective lesion in the outer nuclear layer corresponding with the central hyperreflective point on “en-face” OCT (yellow and blue lines).

A 17-year-old woman was referred with a 7-day history of sudden bilateral blurred vision. She reported being ill for 15 days with fever associated with Marshall syndrome (recurrent symptoms associated with aphthous ulcers, cervical lymphadenopathy, and joint inflammation). Medical and ocular history were unremarkable. She had not inhaled poppers. Best-corrected visual acuity was 20/40 in both eyes. Fundus examination showed a yellowish foveolar hypopigmented lesion without vitritis in both eyes. Autofluorescence measurement and fluorescein angiography were normal (Figures 1(a) and 1(b)). The late phase of ICGA showed a cockade-like hyperfluorescent halo in the macular area (Figures 1(c) and 1(d)) [6]. B-scan SD-OCT images showed disruptions of the line corresponding to the junction between the photoreceptors' inner and outer segments (IS/OS junction) and the RPE inner band. (Figures 1(e) and 1(f)). There was also a large macular hyperreflective lesion involving the external nuclear layer. “En-face” OCT at the level of the IS-OS junction showed a central cockade-like appearance with a hyporeflective center and a hyperreflective border. Moreover, “en-face" OCT showed a punctuate, hyperreflective foveolar lesion in the nuclear layer (Figures 2-3). 

Five days later, visual acuity progressively recovered to 20/20 in both eyes correlating with an improvement in retinal morphology on B-scan SD-OCT. Three weeks later, SD-OCT (B-scan and “en-face”) showed a restored and continuous normal IS/OS layer and RPE inner band. 

### 3.2. Patient 2

A 25-year-old woman presented with a central scotoma in the left eye that had appeared 5 days ago. She had a flu-like syndrome 10 days preceding the visual blurring and she had already undergone an extensive neurologic examination that was negative. She had not inhaled poppers. 

**Figure 4:**
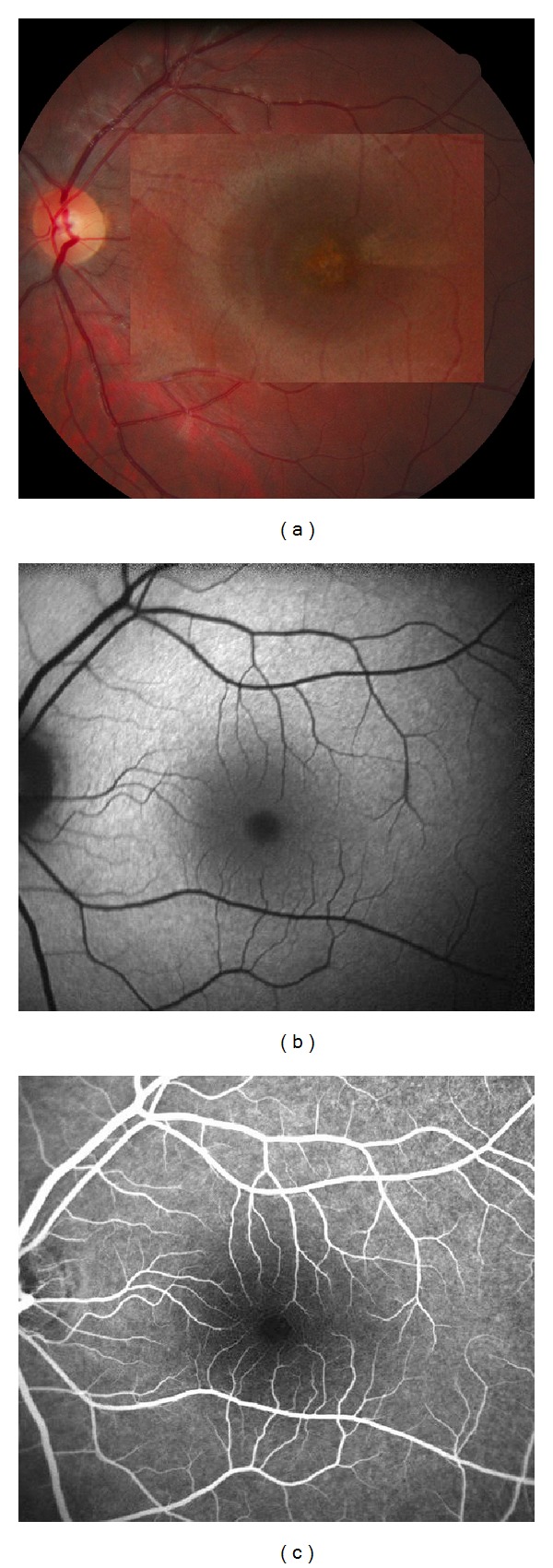
Patient 2, left eye. (a) Color fundus photography showing a yellowish hypopigmented area in the macula. (b) Autofluorescence, and (c) fluorescein angiography, at the initial visit, showing no abnormality.

**Figure 5:**
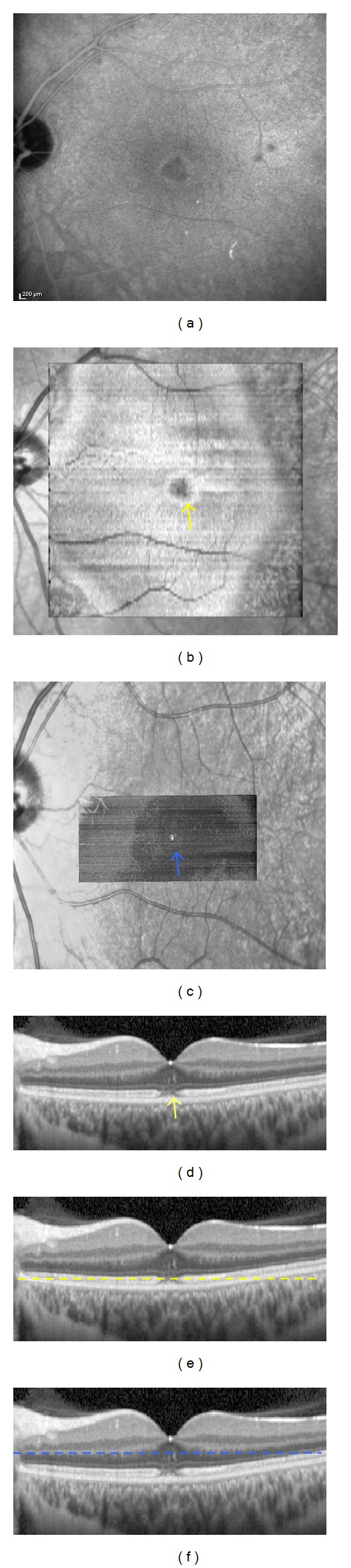
Patient 2. (a) Indocyanine green angiography (20 minutes), ((b)-(c)) “en-face” and ((d)–(f)) B-scan SD-OCT, at the initial visit, showing a cockade-like lesion in the macular area and a hyperreflective lesion between the RPE and IS-OS junction (yellow and blue arrows).

Best-corrected visual acuity in the left eye was 20/32. Color fundus photography of the left eye revealed a yellowish hypopigmented area in the macula. Autofluorescence measurement and fluorescein angiography were normal (Figures 4(a)–4(c)). B-scan SD-OCT revealed a disruption of the inner band of retinal pigment epithelium and IS/OS junction. Further, OCT showed small hyperreflective deposits between the IS/OS junction and the outer nuclear layer. Around this disrupted foveal area, IS/OS junction thickening could be observed as a fusion between the IS/OS junction and the RPE inner band. The late phases of ICGA revealed a hypofluorescent lesion with a hyperfluorescent halo with a cockade-like appearance (Figure 5(a)). “En face” OCT scan at the level of the IS/OS junction demonstrated a hyperreflective halo surrounding the hyporeflective fovea. The halo corresponded to the faint hyperreflective IS/OS junction thickening around the disrupted foveal area. Correlation with late phase ICGA revealed that the “en-face” OCT cockade-like appearance matches the pattern observed on ICGA. Moreover, punctuate hyperreflective foveolar spots corresponding to the central deposits found in the acute phase at the outer nuclear layer level in the B-scan match the hyperreflective point observed on “en-face” OCT (Figures 5(b)–5(f)). 

**Figure 6:**
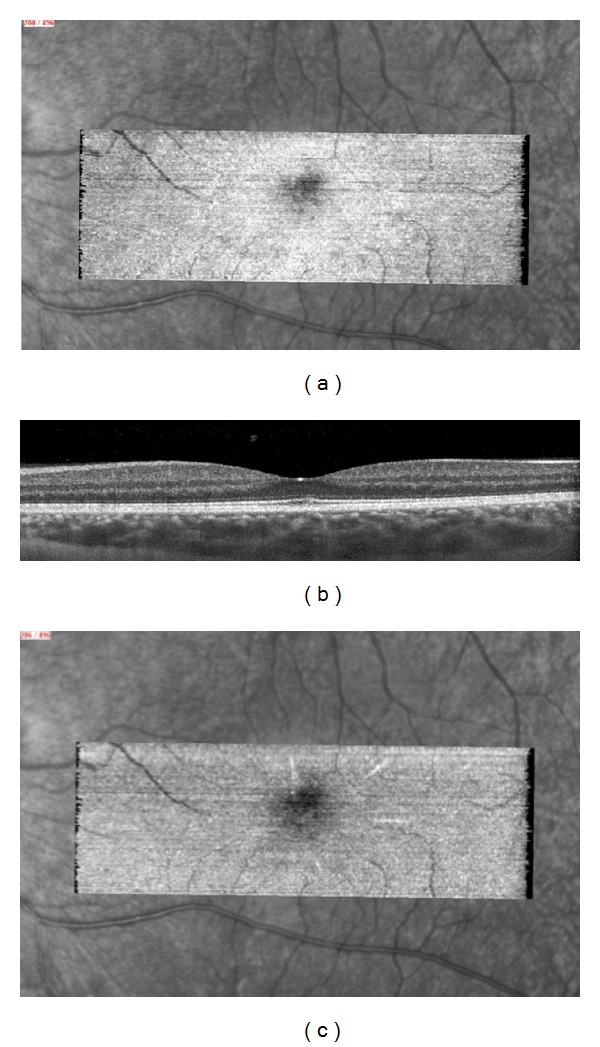
One month later, “en-face OCT” (a) showed an incompletely restored IS/OS line while B-scan (b) seems to be normal. A slight hyperreflective macular star at the ONL level (c) is visible.

One month later, SD-OCT B-scan demonstrated a return to normality while “en-face” scans showed an incomplete recovery of the disrupted central IS/OS junction (Figures 6(a), 6(b)). A transient, slight, hyperreflective macular star could be detected at the outer nuclear layer level (Figure 6(c)). 

## 4. Discussion

The pathophysiology of ARPE is not completely understood. Funduscopy shows a yellowish halo around the fovea, without vitritis. MEWDS (Multiple Evanescent White Dots Syndrome) may have a similar presentation. In both pathologies, the natural history leads usually to complete and spontaneous resolution within weeks. In MEWDS, fundus examination shows differences with discrete perifoveolar dots, very mild vitritis, and, in some cases, papillitis. On fluorescein angiography these dots appear hyperfluorescent and are hypofluorescent on ICGA. There is no cockade-like foveolar lesion as in ARPE. Moreover, EDI-OCT frequently demonstrates choroidal thickening associated with MEWDS lesions [7, 8].

Also, it is important to ask if the patient repeatedly inhaled poppers which may be associated with vision loss due to the disruption of foveal cone outer segments [9]. These lesions can simulate the ARPE lesions that were recently described [6]. Classically, foveal involvement with blurring of the inner pigment epithelium and IS/OS junction is observed on SD-OCT. Small hyperreflective deposits between the IS/OS junction and outer nuclear layer are identified in some cases [10, 11]. Around this disrupted central area, one can frequently observe IS/OS junction thickening as a fusion between IS/OS and RPE inner layers.

C-scan or “en-face” OCT is an interesting technique in order to more precisely characterize the anatomical changes in ARPE [12–15]. The extent of outer retinal damage, mainly at the IS/OS level, is evidenced by “en-face” OCT. The central lesion demonstrates a cockade-like appearance with a hyporeflective center and a hyperreflective border. This cockade-like appearance matches the pattern observed on ICGA. Baillif et al. suggested that this halo on ICGA may be due to a wash-out of ICG in the late phase and staining in the RPE inflammatory cells [6]. C-scan OCT seems to confirm this inflammatory hypothesis with a cockade-like lesion resulting from thickening of the IS-OS junction and the RPE inner band. The macular star depicted in the outer nuclear layer, during the followup, may, correspond to slight retinal exudates due to the hyperreflective inflammatory lesion resolution. Furthermore, punctuate, highly reflective, foveolar lesions, resolving within days, could also be observed. These abnormal findings are observed by that time only with C-scan OCT. During followup, progressive resolution of all the abnormal findings occurs without treatment. C-scan OCT appears to be a more precise and helpful technique for the followup of these lesions.

## 5. Conclusion

Acute retinal pigment epitheliitis refers to a complex disease that resolves quickly without treatment. Outer retinal and pigment epithelial changes observed with multimodal imaging are transient and may disappear quickly in some cases. “En-face” OCT imaging enables the assessment of the extent of structural damage occurring in ARPE. “En-face” OCT enhances its sensitivity, allowing earlier diagnosis of retinal changes and a more reliable followup. Further prospective studies, including more patients, will be necessary to confirm these results.
